# Screen-time Weight-loss Intervention Targeting Children at Home (SWITCH): A randomized controlled trial study protocol

**DOI:** 10.1186/1471-2458-11-524

**Published:** 2011-06-30

**Authors:** Ralph Maddison, Cliona Ni Mhurchu, Louise Foley, Leonard Epstein, Yannan Jiang, Midi Tsai, Ofa Dewes, Ihirangi Heke

**Affiliations:** 1 Clinical Trials Research Unit, University of Auckland, Private Bag 92019, Auckland 1142, New Zealand; 2 Department of Pediatrics, University at Buffalo, G56 Farber Hall, South Campus, Buffalo, NY 14260-0001, USA; 3 Pacific Health, University of Auckland, Private Bag 92019, Auckland 1142, New Zealand; 4 School of Physical Education, University of Otago, PO Box 56, Dunedin 9054, New Zealand

## Abstract

**Background:**

Approximately one third of New Zealand children and young people are overweight or obese. A similar proportion (33%) do not meet recommendations for physical activity, and 70% do not meet recommendations for screen time. Increased time being sedentary is positively associated with being overweight. There are few family-based interventions aimed at reducing sedentary behavior in children. The aim of this trial is to determine the effects of a 24 week home-based, family oriented intervention to reduce sedentary screen time on children's body composition, sedentary behavior, physical activity, and diet.

**Methods/Design:**

The study design is a pragmatic two-arm parallel randomized controlled trial. Two hundred and seventy overweight children aged 9-12 years and primary caregivers are being recruited. Participants are randomized to intervention (family-based screen time intervention) or control (no change). At the end of the study, the control group is offered the intervention content. Data collection is undertaken at baseline and 24 weeks. The primary trial outcome is child body mass index (BMI) and standardized body mass index (zBMI). Secondary outcomes are change from baseline to 24 weeks in child percentage body fat; waist circumference; self-reported average daily time spent in physical and sedentary activities; dietary intake; and enjoyment of physical activity and sedentary behavior. Secondary outcomes for the primary caregiver include change in BMI and self-reported physical activity.

**Discussion:**

This study provides an excellent example of a theory-based, pragmatic, community-based trial targeting sedentary behavior in overweight children. The study has been specifically designed to allow for estimation of the consistency of effects on body composition for Māori (indigenous), Pacific and non-Māori/non-Pacific ethnic groups. If effective, this intervention is imminently scalable and could be integrated within existing weight management programs.

**Trial Registration:**

ACTRN12611000164998

## Background

In New Zealand, more than one third (35.5%) of children and young people aged 5-24 years are overweight or obese [1], which is a level similar to that in other western countries [2]. Childhood obesity is linked to a myriad of health problems, including increased incidence of Type 2 diabetes and cardiovascular disease [3-5]. Given the negative health consequences, reducing the prevalence of overweight among youth is justifiably a public health priority.

Current obesity levels are linked to energy imbalance as a result of reduced energy expenditure and increased energy intake [6]. Sedentary behavior is considered an important determinant of obesity by contributing to this energy imbalance [7]. Derived from the Latin term sedere ("to sit"), sedentary behavior refers to sitting and lying activities that do not increase energy expenditure substantially above the resting level. Operationally, sedentary behavior includes activities that involve energy expenditure at the level of 1.0-1.5 metabolic equivalent units (METs) (one MET is the energy cost of resting quietly) [8]. Sedentary behavior includes a wide range of activities such as screen-based activities, reading and doing homework.

Screen-based activities, such as watching television (TV), playing video games, and using computers are common sedentary behaviors among young people [7], taking up to a total of 3 hours and 40 minutes per day, with a peak between the ages of 9 and 12 years [9]. In New Zealand, only 30% of children and young people aged 10-24 years meet the guidelines for screen time (less than 2 hours/day) [1]. Time spent by children on screen-based activities has been associated with increased risk of obesity [9-13]. Data from a meta-analysis of 52 independent samples (39 studies) found a small but statistically significant association between TV viewing and obesity [13]. TV watching is hypothesized to displace physical activity and reduce energy expenditure [13], as well as increase energy intake due to snack food consumption while watching TV or as a result of food advertising on TV [14,15]. To date, the adverse effect on food intake provides the strongest evidence to date explaining the relationship between TV watching and childhood obesity [14,15]. Other screen activities such as video games are also pervasive. A report from the United Kingdom reported that 91% of children played three or more gaming formats (or platforms), with video console games the preferred choice. For 11-16 year old children, 74% played 3-7 times per week, with an average duration of 1.9 hours per session. Similar results have been reported for 6-10 year olds [16].

To date, most interventions aimed at reducing sedentary behaviors in children have focused primarily on reducing time spent watching TV. Some have been conducted in community settings such as schools [17,18], preschool centers [19] and primary care centers [20], whilst other small studies have been conducted in individual households [21-24]. Generally, results thus far have been encouraging with reductions observed in TV watching, body weight and dietary intake [18,21,23,25]. School-based interventions incorporating electronic TV time monitors (devices that electronically control TV signal and hence availability) as a component have shown particularly striking effects on TV watching and body weight [18,26,27].

A recent trial conducted in the United States (US) [28] randomized 70 children (4-7 years) to an intervention (using TV restriction monitors and education and incentives) to reduce their TV viewing and computer use by 50%. Results showed greater reductions in the targeted sedentary behaviors, standardized body mass index (zBMI), and energy intake in the intervention group compared with those in the control condition. Changes in targeted sedentary behavior mediated changes in body composition. Despite these effects, few studies [29] have examined the effect of an intervention on all screen-based activities (TV, video and computer use).

We will conduct a randomized, controlled trial that will implement and evaluate the effectiveness of a family-based intervention to decrease screen-based sedentary behavior and improve body composition among New Zealand families. A family approach was taken due to the strong influence of parental strategies on children's out-of-school behaviors, including TV watching [29]. The trial will focus on providing primary caregivers with education and training in the use of a wide range of strategies with which to reduce children's sedentary behaviors. One strategy will include the use of electronic devices to reduce TV viewing and video game play.

The primary aim is to determine the effectiveness of a home-based, family intervention to reduce sedentary screen time on overweight children's body mass index (BMI) and zBMI. Secondary aims are to examine the effect of the intervention on body composition, physical and sedentary activities and diet in children and their primary caregiver.

## Methods/Design

### Design

The Screen-time Weight-loss Intervention Targeting Children at Home (SWITCH) trial design is a pragmatic two-arm parallel randomized controlled trial. The study methods below are reported in accordance with the CONSORT guidelines for reporting parallel group randomized trials [30]. Ethical approval for the trial was received from the Lower South Regional Ethics Committee (LRS/10/09/039).

### Participants

Eligible participants are children aged 9-12 years, living in the greater Auckland metropolitan area, who use electronic media (TV, computer, video games) for 15 hours per week or more (according to the primary caregiver), are overweight or obese according to Cole International cut-off criteria [31], can provide written informed assent/consent to participate in study, and speak and understand English. Only one eligible child per household is recruited. Participants are excluded if they have any medical condition that prevents or interferes with their ability to participate in regular physical activity or if they live in more than one household and spend equal time in each household. We are also recruiting one primary caregiver or parent of each child.

Participants are recruited via community contacts, schools, churches, primary healthcare organizations, word of mouth and local advertisements. Interested participants contact the research team via email or telephone and are sent a study pack including participant information and consent forms. Potentially eligible participants and their primary caregiver are screened during a telephone call initiated by the research team. A baseline assessment is scheduled within four weeks of the screening call at the participant's home.

### Outcomes

The primary outcome is change in child BMI and zBMI from baseline to 24 weeks. Secondary outcomes are change from baseline to 24 weeks in child body weight (kg), waist circumference (cm), percentage body fat (%), average daily time spent in moderate to vigorous physical activity (minutes), average daily time spent in sedentary behavior (minutes), dietary intake (average daily energy intake, energy consumed from snack foods, and frequency of soft drink consumption), and perceived enjoyment of physical activity and sedentary behavior. Secondary outcomes for the primary caregiver are change from baseline to 24 weeks in BMI and physical activity (MET-minutes per week).

### Sample size calculation

The target sample of 270 children (135 per arm) will provide at least 90% at 5% level of significance (two-sided) to detect a 0.2 unit difference in change of zBMI (standardized by age and sex) from baseline to 24 weeks between the intervention and control groups, assuming a standard deviation of 0.5. We aim to recruit 1/3 Māori (indigenous), 1/3 Pacific, 1/3 non- Māori/non-Pacific participants (90 children in each subgroup with 45 per arm), which will provide 80% power to detect a 0.3 unit difference in change of zBMI.

### Randomization and blinding

Eligible children are randomized at a 1:1 ratio by computerized central randomization using stratified blocked randomization with variable block sizes to maintain balance across important prognostic factors. Two stratification factors are considered: sex (female and male) and preferred ethnicity (Māori, Pacific and non-Māori/non-Pacific). Participants are informed of their group allocation at the end of the baseline assessment. The project statistician (YJ) oversees the allocation sequence. Research assistants are responsible for enrolling participants and group assignment. Blinding is not possible due to the nature of the intervention and pragmatic study design, but allocation concealment is maintained to the point of randomization.

### Intervention

The intervention is a family-based education program aimed at reducing children's leisure time screen-based sedentary behaviors such as TV watching, computer and video game console use. The intervention is grounded in behavioral economics theory (BET) [32] and social cognitive theory (SCT) [33]. For example, reinforcing value (enjoyment of behavior) and access (availability of activities in the immediate environment), are considered to be key determinants of behavioral choice according to BET. The intervention aims to modify both of these determinants to reduce screen-time. Caregivers praise and reward their children for reducing screen-time (thus modifying reinforcing value). Concurrently, caregivers are encouraged to modify the home environment to make it less screen friendly, such as removing video games from the child's bedroom. Role modeling or observational learning is a key tenet of SCT. In this intervention, caregivers are encouraged to be positive role models for their children and to modify their own screen behaviors to reflect the behaviors they would like their children to perform.

The focus of the intervention is to train the primary caregiver to initiate changes in the home environment to facilitate a reduction in children's leisure-time screen activities. Trained culturally appropriate research assistants educate the primary caregivers regarding the intervention content during a one-hour face-to-face meeting at their home, within two weeks of the baseline assessment. The content of the intervention was based upon work by Epstein and colleagues in the US [28] and adapted for the New Zealand sociocultural context. Further details of the intervention components are provided in Table 1.

**Table 1:** Screen-time Weight-loss Intervention Targeting Children at Home (SWITCH) trial intervention components

Strategy	Explanation of strategy to caregiver	Tips provided to caregiver	Examples	Theoretical basis
Praise	Praise involves using words and actions to tell your child that you liked what he or she did. Praising your child's behavior will not only make them feel good, it will also make them want to repeat that behavior.	Tips for using praise: Observe your child to catch them being good. Give the praise immediately or as soon as possible after the desired behavior. Praising before the behavior, or too long after, will not be effective. Be specific about what you praise. Tell your child exactly what they have done that you like. Be consistent with your praise. What is good behavior today should be good behavior tomorrow.	"I am so proud of you for finding other things to do than watch TV. Great!" "I am very pleased that you stuck to your budget and only watched TV for 30 minutes today."	Behavioral Economics Theory - modification of reinforcing value

Positive reinforcement	Positive reinforcement involves giving a reward to your child once a desired behavior has been performed. A reward can be as simple as praise, or as involved as a family outing. After your child is given the reward, he or she will want to do the good behavior again.	Be consistent with reinforcement. Always give the reward you have promised. Do not give the reinforcer if your child has not earned it. A reinforcer only strengthens behaviors if it is earned.	You didn't play any video games today. I told you that if you did this we would play catch. Let's go!"	Behavioral Economics Theory - modification of reinforcing value

Environmental control	You can change the home environment to reduce the number of cues that prompt screen time. You can also make changes that increase the number of cues or opportunities to do other alternative activities.	Try to break the habit of your child coming home from school and watching TV or using the computer. You could have a rule that states when you child gets home they can have a snack, but then must do something other than watch TV for half an hour.	Remove TV or video games from your child's bedroom. Put video games in hard to reach places. Hang a sign over the TV during times your child plans not to watch. Have toys easily available, or an area set up where your child has everything they need to do homework.	Behavioral Economics Theory - modification of access

Budgeting and self-monitoring	Research assistant connects the Time Machine to the two devices most commonly used by the child. Research assistant explains to the caregiver how these devices can be used to budget the amount of time the child spends on each device.	Cut down your child's use gradually. For the first week, start by decreasing their time on the TV or computer by 10% compared to their usual amount. Reduce this by 10% per week. Agree on a budget by discussing with your child. Help them plan their screen-time for the week to ensure they don't go over the budget. This will help them to "buy-in" to the idea of having a budget and may help them stick to it. Post the budget in a visible place, like on the fridge.		Behavioral Economics Theory - modification of access

Positive role modeling	You can help your child meet their goals for reducing screen-time by supporting their efforts with your own and other family members' behaviour. Many families use the TV for background noise even though they aren't really paying attention to it. If you want your child to stick to their screen-time budget, you can help by turning off the TV when you are not watching it.	Just as modeling is a powerful way to teach your child good behaviour it can also create or encourage poor habits. Modeling poor behaviours in front of your child may result in him or her repeating those same behaviours. Involve the whole family. It will be hard for your child to stay away from the TV if the rest of the family is watching it. The old saying, "Do as I say, not as I do," will not work. Your child will not understand why it is okay for you to watch TV while he or she cannot.	Eat together as a family without the TV on You turn off the TV in the evening and read a book/magazine instead. Older siblings play with your child together rather than playing video games.	Social Cognitive Theory - modeling

Alternative activities	Research assistant provides caregiver with an activity pack and explains that this can be used to provide alternatives to sedentary activities. They can also be used as rewards outlined in the positive reinforcement strategy. Emphasize to caregivers that less screen-time means more time for doing homework.	Contents of activity pack: Activity cards, Tennis ball Elastics, Yo-yo, Colored pencils, Chalk, Playing cards, Checkers, 2 m rope with loops at end, Stickers SWITCH magnet Te Reo octopus game (Māori)	After the activity pack has been given to the family, the research assistant teaches the caregiver and child a couple of games using items from the activity pack.	Behavioral Economics Theory - modification of access

Time Machine (Family Safe Media, Park City, US) TV monitoring devices are used as a tool to assist families to budget their TV watching time. The Time Machine connects to a TV via the coaxial cable connection on the TV or the Radio Corporation of America (RCA) connections from other video sources (digital video disk [DVD] player, video game console, etc.), but cannot be connected to a computer. The cables are secured and locked to the back of the device to prevent tampering. Parents are given the keys to access the cables. Such devices have been successfully used in research in the US [28] to budget TV time, and this device was used in a New Zealand pilot study [22]. Because the Time Machine controls the amount of time the TV can be switched on, parents and caregivers can use this device to help budget TV watching time and set screen free times during homework or bedtime hours. Each Time Machine comes with 30 tokens, each allowing 30 minutes of viewing time. The digital display notifies the user when the time is almost up. At the face-to-face meeting, a research assistant connects the Time Machine to the two devices (TV, DVD player or video game console) most commonly used by the child.

Participants in the intervention group also receive an activity pack containing suggestions and options for alternatives to screen-based activity. The packs (see Table 1) include coloring pencils, a length of rope, playing cards, a tennis ball for playing handball or similar games, and activity cards, which describe simple games or activities to play. Māori-specific board games and instructions for traditional Māori games are also offered to all participants.

Throughout the study, brief (one page) tailored monthly newsletters are delivered to primary caregivers outlining additional strategies for reducing screen-based sedentary activities. There are different versions of each newsletter for Māori, Pacific and non-Māori/non-Pacific families. While the content of the newsletters is the same for each group, the presentation and language used differs. A website http://www.switchtrial.co.nz is also provided to support the intervention content, and includes the monthly newsletters in electronic format, additional tips and information, and links to community-based activity programs. The website content can only be accessed by participants in the intervention group via a unique login and password. At 12 weeks the primary caregiver is contacted by telephone to confirm their contact details, assess the fidelity of the intervention, and record any serious adverse events. Fidelity is assessed by asking caregiver's specific questions about the number and type of strategies used.

### Control

The control group is asked to continue with their normal behavior (no change). The primary caregiver is contacted at 12 weeks to confirm their contact details and record any serious adverse events. At the end of the study, the control group is offered the intervention content.

### Procedure

Assessments are undertaken at participants' home at baseline and 24 weeks post-randomization. An appointment is made at an agreed time to undergo assessment. At the baseline assessment, the research assistant obtains written assent/consent. Physical measurements of the child's height, weight, waist circumference and body composition (bioelectrical impedance) are conducted prior to completing self-reported measures of physical activity, sedentary behavior, diet, and measures of enjoyment of physical activity and sedentary behavior. Height and weight is measured for each primary caregiver prior to their completing a seven day recall physical activity questionnaire. Following the baseline assessment, participants are randomized to intervention or control. Intervention group participants receive the intervention components described above. Both groups receive a follow-up phone call at 12 weeks. At the 24 week assessment, all measurements are repeated.

### Measures

#### Anthropometric measures

Anthropometric data are measured using standard practices [34]. Height is measured to the nearest 0.1 cm with a stadiometer (Seca, 214, Hamburg, Germany) and weight is measured to the nearest 0.1 kg with a digital scale (Tanita, UM-070, Illinois, US) according to standard procedures [34]. For both height and weight, two measures are taken. A third measurement is performed if differences of 0.1 cm and 0.1 kg respectively are observed between the first and second measurements. The mean of two measurements or the median of three is used for analysis. BMI is calculated as weight divided by height squared. zBMI is calculated at each time point using age and sex-specific BMI data from the 2002 New Zealand National Children's Nutrition Survey [35]. Waist circumference data are collected using a flexible non-elastic tape measure. The tape measure is placed around the participant's waist at the level of the umbilicus. Two measurements are taken, or a third if the difference between the two measurements is greater than 0.1 cm. The mean of two measurements or the median of three is used for analysis.

#### Child fat mass, fat free mass and percent body fat

Bioelectrical impedance (BIA) is used to estimate children's percentage body fat, fat mass, and fat-free mass [36] using the ImpediMed DF50 Bioimpedence Monitor (Queensland, Australia). Prior to testing, participants are required to lie quietly in a supine position and kept warm to ensure good circulation. The participant is instructed to lie with their legs slightly apart and hands resting next to the body palms down.

An alcohol wipe is used to remove excess oil and electrodes are placed on the right wrist between the two protruding bones, on the dorsal surface of the right hand 1 cm proximal to the middle knuckle, on the right ankle between the medial and lateral malleoli and on the dorsal surface of the right foot, 1 cm proximal to the metatarsal phalangeal joint of the second toe. The yellow and red leads are connected to the electrodes on the participant's wrist and hand respectively, and the blue and black leads are connected to the electrodes on the participant's ankle and foot, respectively. During testing, participants are required to lie still and not talk. Two separate readings are taken from each participant. Output values of impedance, phase, resistance and reactance are recorded. The Rush equation [37] is used to calculate fat mass, fat free mass and percentage body fat for all participants.

#### Child self-reported physical activity and sedentary behaviors

Children's physical activities and sedentary behaviors are recalled using the Multimedia Activity Recall for Children and Adolescents (MARCA) [38]. The MARCA is a computerized 24 hour recall use-of-time tool. It consists of three modules, a previous day activity recall, a compendium of child-specific energy costs, and an analytical module. The MARCA asks children and young people to recall their previous day's activities in time slices of five minutes or more, and participants can recall a school day or another day (weekend, holiday, or day off from school). Previous research has shown the MARCA to have a same-day test-retest reliability of r = 0.84-0.92 for major outcome variables [sleep, moderate to vigorous physical activity (MVPA), physical activity level (PAL) and screen time (the number of minutes spent watching TV, playing videogames and using a computer)], and validity with reference to accelerometry of r = 0.45 for PAL [38].

The MARCA contains a drop down list of everyday activities that participants can choose from, e.g., 'brushing teeth', 'dressing and undressing', 'walking' and 'soccer (field/indoor)'. The participant chooses an activity and indicates the duration of the activity, which is then recorded on the MARCA. Each activity has an associated energy cost (METs). For the purpose of this trial, participants are asked to recall the two previous days (48 hours) of activity at each assessment. When two activities are reported simultaneously such as doing homework while watching TV, the following hierarchy is used: physical activity, screen time, active transport, passive transport, and other activities. For example, watching a DVD while being driven to a destination would count as watching a DVD, while watching TV while running on a treadmill would count as running. Average daily time (minutes) spent in physical activity and sedentary behavior is derived for analysis. Average daily time (minutes) spent in screen and non-screen sedentary behaviors is also derived.

#### Adult self-reported physical activity

The International Physical Activity Questionnaire (IPAQ) [39] is a standardized self-report measure used to estimate habitual physical activity in adults. The long-form of this questionnaire is used for this trial. The IPAQ assesses time spent walking, doing moderate-intensity and vigorous-intensity activity within the domains of work, transportation, domestic and gardening (yard) activities, and leisure-related activities. It also assesses time spent sitting. Activities are recalled for the previous seven days. The IPAQ has been shown to be a reliable (same-week reliability rho = 0.8) and valid (rho = 0.3 against accelerometry) tool for assessing physical activity [39]. The weighted MET-minutes per week are calculated as duration × frequency per week × MET value, which are summed across activity domains to produce a weighted estimate of total physical activity from all reported activities per week.

#### Child psychological variables related to physical activity and sedentary behavior

Psychological variables are measured to determine their potential mediating effect. Perceived enjoyment of physical activity is assessed using the 14 item Physical Activity Enjoyment Scale [40], adapted for use in adolescents in 2001 [41]. Participants rate their agreement with statements (e.g., "When I am active I enjoy it") on a five point Likert scale. Scores are summed, with higher scores indicating a higher level of enjoyment.

Perceived enjoyment of sedentary behaviour is assessed using a scale adapted from Salmon et al [42]. Participants are required to rate their level of enjoyment of nine sedentary behaviours (e.g., TV viewing, sitting and socializing, reading) on a five point Likert scale. The original scale was used developed for adults; however it was adapted for this study to be appropriate for children. Scores are summed and averaged across the nine items, with higher scores indicating a higher level of enjoyment.

#### Child dietary intake

A semi-quantitative food frequency questionnaire (FFQ) will be used to collect information on frequency of consumption of common New Zealand foods. The FFQ was developed for use in the New Zealand Children's Nutrition Survey [35] and has similar repeatability to child or adolescent FFQs used in other countries [43]. The FFQ comprises a list of 104 commonly consumed food items (including illustrations) and respondents are asked to indicate how often they have eaten these foods over the past four weeks. For each item of food (i.e., bananas, raw) the respondent is asked to tick one of the following boxes, never or less than once a month; 1-3 times a month; 1-2 times a week; 3-4 times a week; 5-6 times a week; or 2 or more times a day. Photos of standard portion sizes on a dinner plate are also provided next to each item of food for respondents to choose the portion size that most closely approximates their usual intake amount. Total daily energy intake will be estimated, as well as energy intake from snack foods, and consumption of specific food items (e.g., snack foods and sugar-sweetened beverages).

#### Post-trial interview (intervention group)

Primary caregivers in the intervention group are asked to complete a semi-structured interview to obtain feedback on the intervention. The interview is a mixture of closed and open questions to identify the components of the intervention they found most helpful, the strategies they used, and suggestions for improvement in the future. They are also asked to provide information on any additional strategies they implemented.

### Statistical analysis

A pre-specified analysis plan outlines the detailed statistical methods. Treatment evaluation will be performed on the principle of intention to treat, using data collected from all randomized participants. An analysis of covariance (ANCOVA) regression model will be used to evaluate the main treatment effect on the primary outcome between the two treatment groups, adjusting for baseline measures, important demographics and other potential confounding factors (if they are statistically significant at 5% level). A similar approach will be used for secondary outcome measures. Multiple imputation methods will be applied to missing data (if any) for the primary outcome only. No imputation will be used for other secondary outcomes. Statistical analyses will be performed using SAS version 9.2 (SAS Institute Inc. Cary NC) and R version 2.11 (R Foundations for Statistical Computing). All statistical tests will be two-tailed and a 5% significance level maintained throughout the analyses.

## Discussion

The family-based intervention described in this paper is the first of its kind in New Zealand and is one of few interventions targeting screen-based sedentary behaviors in the home environment. The study is focused on training parents or caregivers to facilitate change in the home, and if effective this approach in imminently scalable through existing weight loss programs and parent education services.

This research builds on recent work by Epstein and colleagues [28] by adapting the intervention for a New Zealand setting and for ethnic minority groups (Māori and Pacific). The study is adequately powered to investigate consistency of effects for these groups. Few studies have examined the effect of interventions to reduce sedentary behavior in minority groups [44]. In this study, we aim to recruit equal numbers of Māori, Pacific, and non-Māori/non-Pacific participants, and proactive steps were taken to maximize recruitment in these populations. The study investigator team includes both Māori and Pacific researchers, whom have been involved in the study design, development of the intervention, and consultation on cultural issues relevant to the conduct of the research. We also have specialized Māori and Pacific research assistants who are involved in the recruitment of these populations and deliver the intervention to Māori and Pacific families.

The SWITCH study provides an excellent example of a pragmatic trial, in which the study is designed to maximize the ecological validity of the findings [45]. Pragmatic trials are beneficial because they provide an indication of the effectiveness of an intervention in a real world setting compared to standard or usual care [46]. However, because of their pragmatic nature these trials are often not blinded, which affects internal validity. To minimize this, in the current trial we use an objective measure for the primary outcome, BMI. For this type of intervention, we believe that the pros of using a pragmatic approach outweigh the cons.

Results of the SWITCH study are due in 2013, and will be disseminated widely to relevant community and government organizations, as well as published in academic journals. In the meantime, it is hoped that the issues discussed provide guidance to those undertaking similar trials with children.

## List of abbreviations

ANCOVA: Analysis of Covariance; BET: Behavioral Economics Theory; BMI: Body Mass Index; CONSORT: Consolidated Standards of Reporting Trials; DVD: Digital Video Disk; FFQ: Food Frequency Questionnaire; IPAQ: International Physical Activity Questionnaire; MARCA: Multimedia Activity Recall for Children and Adolescents; MET: Metabolic Equivalent; MVPA: Moderate-to-Vigorous Physical Activity; PAL: Physical Activity Level; RCA: Radio Corporation of America; SWITCH: Screen-time Weight-loss Intervention Targeting Children at Home; TV: Television; SCT: Social Cognitive Theory; US: United States; zBMI: Standardized Body Mass Index

## Competing interests

The authors declare that they have no competing interests.

## Authors' contributions

Principal responsibility for study design and conduct was assumed by RM. RM, CNM, LF, LE, MT, IH and OD contributed to the development of the intervention and study design. YJ undertook sample size calculations and designed the statistical analysis plan. LF and MT are involved in the study design, data collection and overall project management. RM drafted the manuscript. All authors read and commented on drafts and approved the final manuscript.

## Pre-publication history

The pre-publication history for this paper can be accessed here:

http://www.biomedcentral.com/1471-2458/11/524/prepub
